# Molecular Characterization of a Human Matrix Attachment Region Epigenetic Regulator

**DOI:** 10.1371/journal.pone.0079262

**Published:** 2013-11-14

**Authors:** Salina Arope, Niamh Harraghy, Milos Pjanic, Nicolas Mermod

**Affiliations:** Laboratory of Molecular Biotechnology, Institute of Biotechnology, University of Lausanne, and Center for Biotechnology UNIL-EPFL, Lausanne, Switzerland; University of Texas Southwestern Medical Center, United States of America

## Abstract

Matrix attachment regions (MAR) generally act as epigenetic regulatory sequences that increase gene expression, and they were proposed to partition chromosomes into loop-forming domains. However, their molecular mode of action remains poorly understood. Here, we assessed the possible contribution of the AT-rich core and adjacent transcription factor binding motifs to the transcription augmenting and anti-silencing effects of human MAR 1–68. Either flanking sequences together with the AT-rich core were required to obtain the full MAR effects. Shortened MAR derivatives retaining full MAR activity were constructed from combinations of the AT-rich sequence and multimerized transcription factor binding motifs, implying that both transcription factors and the AT-rich microsatellite sequence are required to mediate the MAR effect. Genomic analysis indicated that MAR AT-rich cores may be depleted of histones and enriched in RNA polymerase II, providing a molecular interpretation of their chromatin domain insulator and transcriptional augmentation activities.

## Introduction

In mammalian cells, gene expression is tightly regulated but flexible. This flexibility stems from alterations in chromatin structure that allow gene expression to be modified in response to cellular requirements or extra-cellular signals [1]–[3]. Upon integration into eukaryotic genomes, a transgene becomes subjected to the regulation of the environment in which it finds itself, which frequently is heterochromatin, a transcriptionally unfavourable region. Shielding the transgene from the environment of the integration site is therefore of great interest from a biotechnology and gene therapy perspective, as it should allow sustained and correctly regulated expression of the transgene.

The discovery that chromatin may be segregated into topologically constrained domains separated by genetic boundary elements such as scaffold or matrix attachment region (MAR) [4]–[10], and that MARs may also act as insulator elements, halting the spread of heterochromatin and preventing transgene silencing [11], [12], gave rise to their use in mammalian expression vectors. By creating an independent chromatin domain, the MAR would shield the transgene from the repressive effects associated with heterochromatin, which has been termed the “anti-silencing” effect [13]–[19]. One human MAR termed MAR 1–68 was shown to decrease the probability of transgene silencing, as well as to increase the probability of switching from an inactive promoter to a transcriptionally active state [20]. Another attractive feature of MAR elements is the ability of some elements to augment transgene expression [13], [16], [17], [19], [21], [22]. This was linked to the ability of some MARs to increase transcription initiation, to augment the number of transgenes integrated into the genome, and/or to possibly target the transgene to transcriptionally favourable regions [11], [22], [23].

Although the function of MARs appears to be evolutionary conserved, attempts to dissect such elements and ascribe functions at the primary sequence level have generally been unsuccessful. MARs typically contain AT-rich elements that consist of regions of alternating A and T several hundred bases in length, or that contain dispersed short AT-rich patches that have a strong potential for base un-pairing when subjected to superhelical strain [24]. Thus, the secondary structure of MARs was proposed to be mainly responsible for their functional activities. Studies of the biophysical and biochemical properties of MARs have revealed that their propensity to unwind and undergo strand separation under stress might be key features that define MARs, when considered in conjunction with binding to the nuclear matrix and the propensity to form a curved structure [22], [24]–[26]. However, defining discrete elements or consensus sequences that may mediate the MAR activity has been difficult. For instance, dissection of the chicken lysozyme MAR revealed that binding of the MAR to the nuclear matrix was not sufficient to enhance transgene expression and that increased transgene expression was in part copy-number dependent [19], [22]. However, the elements required for increased transcription and copy number were not identified [27]. A subsequent bioinformatics characterization of human MAR elements suggested their organization in several distinct components, including an AT-rich core sequence mediating particular structural properties such as bent DNA as well as flanking sequences enriched in binding motifs for specific transcription factors [22]. This combination formed the basis of an in silico program that allowed the screening of the human and mouse genomes for potent MAR elements, which resulted in the identification of new human elements that strongly increased gene expression and stability, such as MAR 1–68 and MAR X-29 [22], [28].

A constraint to the usage of MAR elements remains their size, sometimes of several kb, which limits their use, for instance in gene or cell therapy vectors. Defining the elements essential for transcription augmentation and anti-silencing may be of interest, for instance to allow for shorter constructs to be generated. In this study, we therefore attempted to identify functional elements of MARs and to assess their molecular action on chromatin structure. Our results imply that the MAR transcriptional augmentation and anti-silencing activities result from the combination of AT-rich sequences that may be depleted in histones but enriched in RNA-polymerase II, as well as from transcription activating protein binding sites. These results provide a molecular explanation for the epigenetic insulator and transcriptional augmentation activities of MARs.

## Results

### MAR Elements Lower the Occurrence of Silent Cells and Augment Expression Levels

Potent MAR elements were previously identified by scanning the human genome using the SMARScan MAR-prediction program, yielding DNA elements that could potently enhance transgene expression [22]. The program relied on the identification of AT-rich sequences that were predicted to correspond to bent or bendable DNA structures surrounded by the occurrence of predicted bindings sites for transcription factors known to associate with MAR sequences. However, functional evidence of the potential contribution of such elements, or of combinations thereof, to MAR activity has been lacking. Here, we sought to characterize the molecular actions and DNA sequences or elements that support the MAR function to mediate elevated transgene transcription and/or to reduce the percentage of non-expressing or silenced cells.

**Figure 1:**
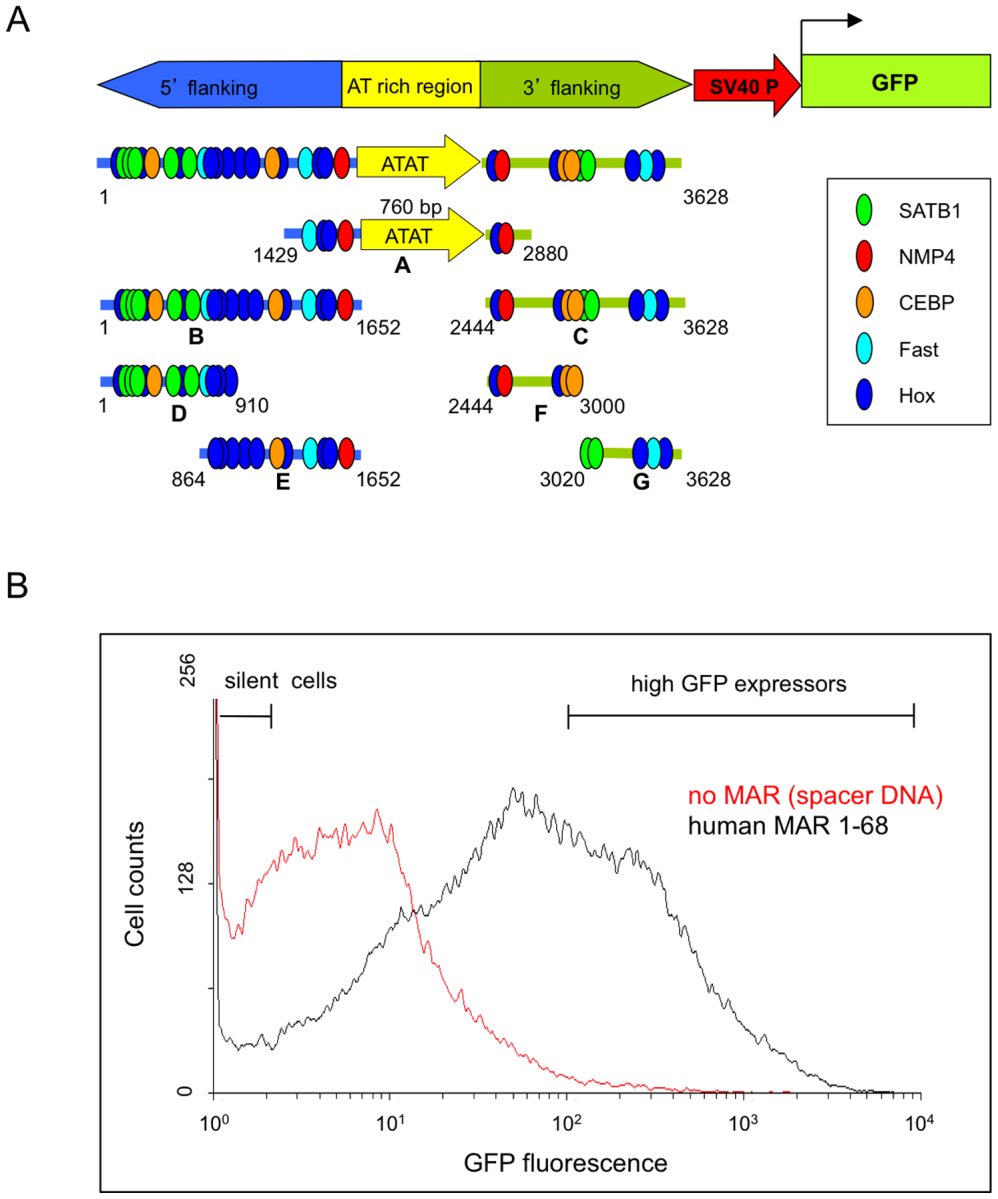
Schematic representation of MAR 1–68 subdomains and illustration of its anti-silencing and transcriptional effects. (A) Schematic diagram representing the full-length human MAR 1–68 and its series of sub-fragments, cloned upstream of a minimal SV40 promoter and EGFP reporter gene. The 3.6 kb MAR 1–68 was subdivided into three regions: The MAR 1–68 “extended AT core” region encompassing the AT dinucleotide-rich sequence (yellow box, labelled A), its 5′ (blue, labelled B) and 3′ (green, labelled C) adjacent regions. Putative transcription factor binding sites for the SATB1, NMP4, CEBP, Fast and Hox transcription factors are illustrated by ellipses. The 5′ and 3′ flanking regions were further divided in portions comprising nt 1–910 (labelled D), nt 864–1652 (E), nt 2444–3000 (F) and nt 3020–3628 (G). (B) A typical flow cytometry profile of CHO DG44 cells stably co-transfected with the GFP expression vector containing full-length human MAR 1–68 (black line) or control spacer DNA (no MAR, red line) and with a neomycin resistance plasmid. 10^5^ cells were subjected to flow cytometry analysis for GFP expression after 2 weeks of nemomycin selection. Cells displaying background fluorescence (silent cells) or high GFP expression levels are as indicated.

**Figure 2:**
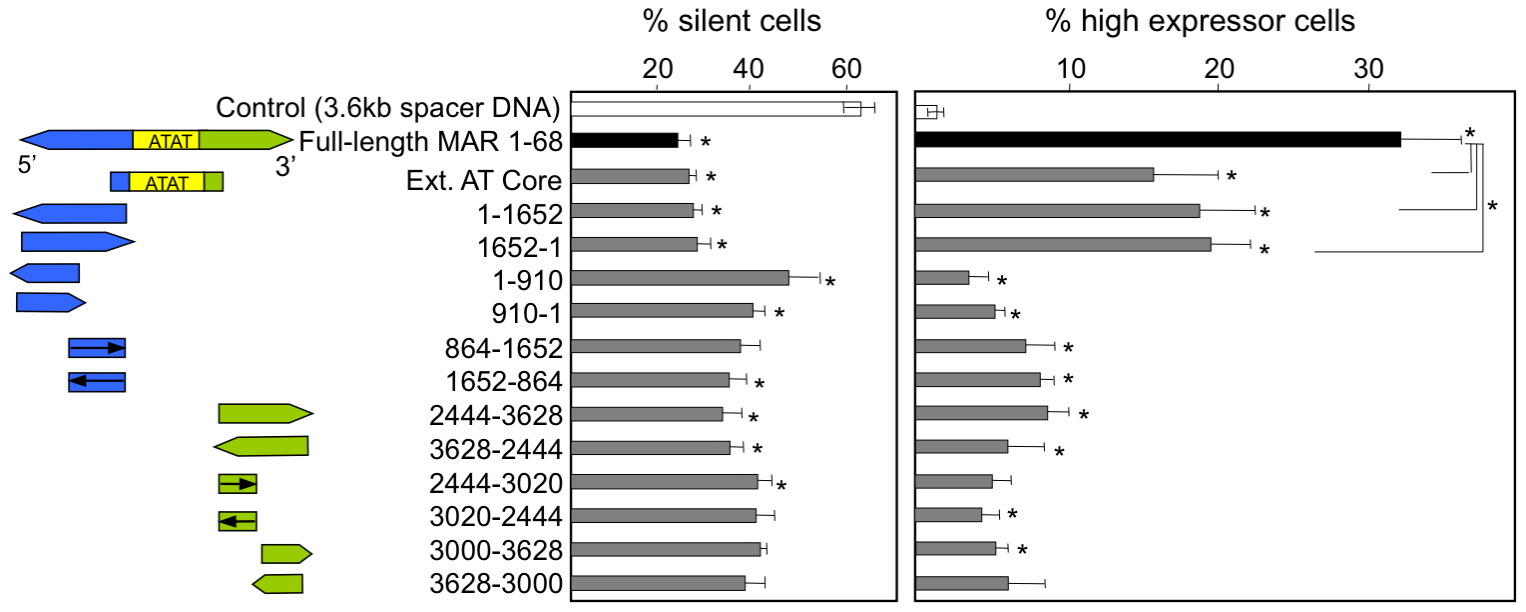
Identification of the portions of MAR 1–68 that contribute to the anti-silencing and transcriptional effects. The AT core extended region of the MAR 1–68, as well as a series of sub-fragments of the 5′ and 3′ flanking regions, were cloned upstream of the EGFP reporter gene in both orientation and analyzed for their effects on GFP expression levels. Constructs containing the full-length MAR 1–68 or a control spacer DNA cloned upstream of the EGFP reporter gene were also transfected as controls. GFP fluorescence was measured by flow-cytometry on polyclonal cell pools obtained after 2 weeks of antibiotic selection following transfection, and the proportion of silent and of high expressor cells were scored as illustrated in Fig. 1B. Results illustrate the mean and standard deviation of 3 independent experiments. Significant differences relative to the corresponding control construct containing spacer DNA of the same size, as illustrated in Suppl. Fig. S3, are indicated by stars above each bar, whereas line-associated stars indicate significant differences with constructs containing the full length MAR 1–68 or its extended core (Student test, P<0.05).

The effect of one of the most potent MAR elements, namely human MAR 1–68, was assessed after insertion upstream of a GFP expression cassette (Fig. 1A). Stable transfections of CHO cells were performed, and the flow cytometry profiles of antibiotic-resistant cell pools were analyzed. Analysis of populations generated without the MAR showed a multimodal distribution of the polyclonal population. One sub-population of cells had relative fluorescence units below 10, which corresponds to the background fluorescence of non-transfected cells (data not shown), whereas another sub-population had fluorescence levels of lowly expressing cells (Fig. 1B). In the presence of the full length MAR 1–68, an increased number of cells expressed detectable levels of GFP, and the transcription levels were increased, yielding subpopulations of medium or high GFP-expressing cells. Quantification of the proportion of cells in each category indicated two effects of the full-length MAR (Fig. 1B and 2). The first effect is the decrease of the percentage of silent cells that do not display significant GFP fluorescence above background, which corresponds to an anti-silencing effect. The second effect is a concomitant increase of the expression level in the broad peak of GFP-expressing cells, referred to as the transcriptional augmentation effect. These results are in agreement with previous studies that indicated that MAR 1–68 can prevent long-term epigenetic silencing effects and increase the transgene transcription rate [22], [23].

Previous work with MAR 1–68 indicated that it acts in part to increase the number of transgene copies that stably integrate into the cell genome [23]. The contribution of the increased copy number relative to transgene expression was assessed on polyclonal populations grown for over 1 month in culture after stable transfection, to ensure stable expression [20]. Cells were sorted according to GFP fluorescence into 4 subpopulations, corresponding to no, low, medium and high GFP expression (Fig. S1A). DNA and RNA were extracted from each sample, and GFP transgene copy number and mRNA were analyzed by quantitative PCR. This revealed a high degree of correlation between transgene copy number and gene expression (Fig. S1B). Highly expressing cells generated with MAR 1–68 had a significant 16-fold increase of the transgene copy number relative to silent cell subpopulations. Furthermore, within the peak of expressing cells, the level of fluorescence correlated well with the transgene copy number. When the GFP mRNA levels of the high-expressor cells were directly compared to those of silent cells, mRNA levels were increased by approximately 200-fold and 2200-fold when comparing highly expressing to silent cells generated without or with the MAR, respectively (Fig. S2). This showed that expression increased more than copy number, especially in presence of the MAR 1–68, as expected from its transcriptional activation effect. When mRNA levels were normalized to the relative transgene copy numbers, expressing cells generated with the MAR had a 160-fold increase of GFP mRNA levels over the control, whereas MAR-devoid cells displayed lower levels of GFP mRNA. Overall, we concluded that the variability of expression levels results in part from variations in the number of integrated transgene copies, and that the MAR acts in part by increasing the frequency of cells that integrated a high number of transgene copies, and in part by increasing the rate of transcription per transgene copy in agreement with prior observations [20].

Three structurally distinct regions of MAR 1–68 could be identified as a central AT-rich core region and the 5′ and 3′ flanking regions containing transcription factor binding motifs for the NMP4, SATB1, Fast 1 and CEBP transcription factors (Fig. 1A). In contrast, the core region was enriched in the (ATAT)_n_ microsatellite, but it was devoid of any such binding motif. To assess whether functional properties of MAR 1–68 may be ascribed to these sequences, the full length element was dissected into smaller fragments that were inserted in the MAR-devoid expression vector. The AT-rich core as well as the full-length 5′ flanking region were found to be the most potent among the three MAR 1–68 portions in mediating the anti-silencing and increased transcription effects. These two MAR fragments were of nearly equal strength as that of the full-length MAR 1–68 in terms of the anti-silencing effect, reducing the proportion of non-expressing cells to 25–30% of the total polyclonal cell population, whereas inclusion of non-MAR control sequences had little effect (Fig. 2 and S3). However, their transcriptional activation ability was approximately half that of the full-length MAR 1–68, corresponding to about 15–20% of the total cell population displaying high GFP fluorescence. The full-length 3′ flanking region did increase expression and decrease silencing significantly when compared to cells transfected with the corresponding pEGFP control plasmid. However, transcriptional activation was reduced when compared to the 5′ flank and AT-core extended regions of the MAR. The orientation of the 5′ and 3′ flanking sequence did not affect GFP expression. Smaller portions of the MAR 5′ flanking regions exhibited reduced but often significant transcriptional activation and anti-silencing effects. Fragments of the 3′ flanking sequence exhibited low or insignificant anti-silencing transcriptional activation effects. Overall, these results implied that multiple sequences present in the 5′ and 3′ flanking sequences may contribute to the MAR effects.

**Figure 3:**
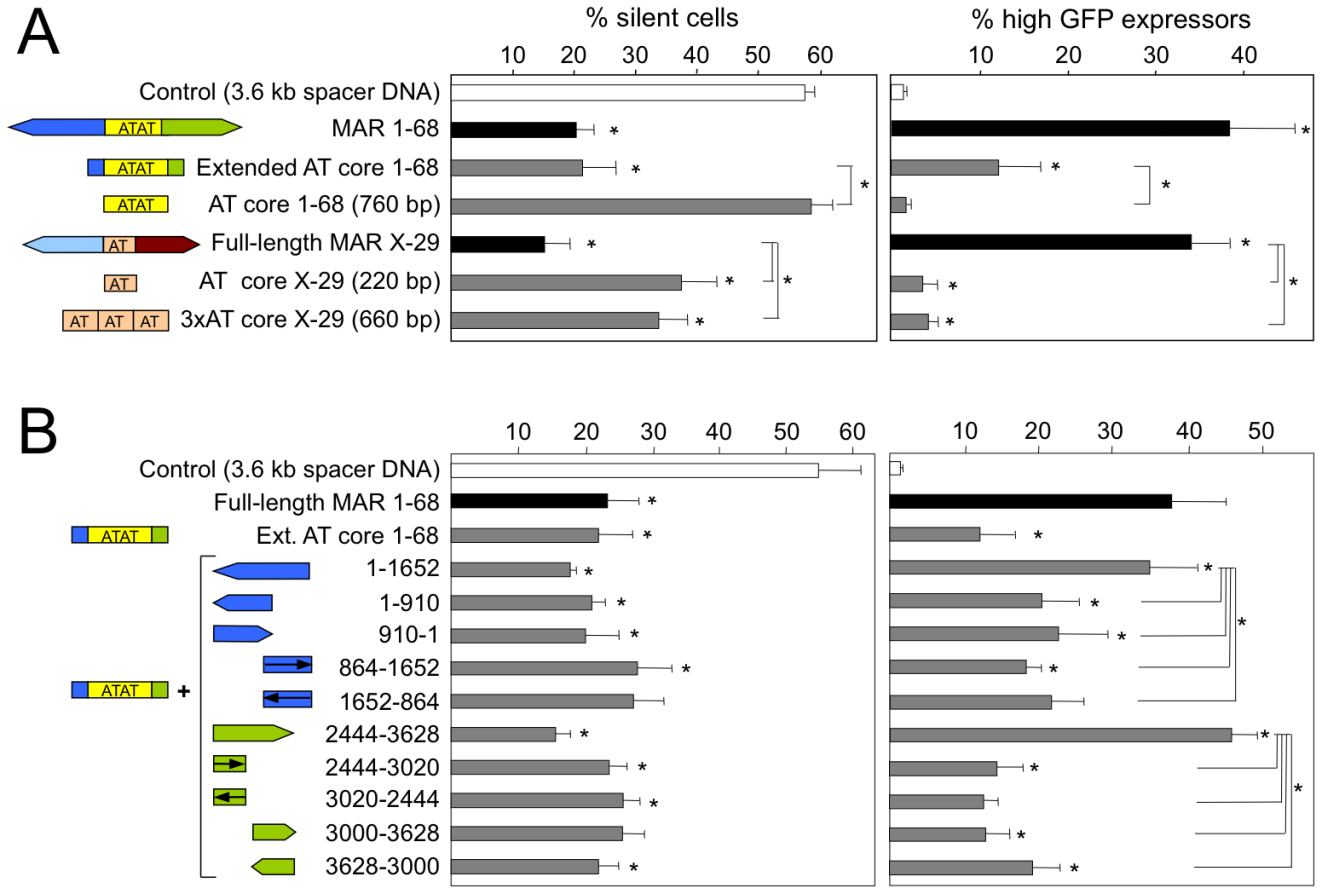
Relative contribution of MAR AT-rich cores and flanking sequences to the anti-silencing and transcriptional effects. The contribution of the AT rich DNA sequences of MAR 1–68 and X-29 alone (A), or combinations of the MAR 1–68 core with portions of its flanking sequences (B), were assessed for their anti-silencing and transcriptional augmentation activities as described in the legend to Fig. 2. An oligomeric form of the X-29 AT-rich region, consisting of three tandem repeats, was also analyzed. Results represent the mean±SD of 3 independent experiments and the statistical analysis are as for Fig. 2.

We next investigated whether isolated MAR AT-rich sequences may be sufficient to drive transgene expression and inhibit transgene silencing. The AT-rich core sequences of 760 bp in length from MAR 1–68, composed almost exclusively of A, T and C bases on one strand, was isolated from the non AT-rich sequences. Another AT core sequence of 220 bp in length that contains longer stretches of alternating A and T separated by occasional G and C bases was obtained from another potent human MAR from the X chromosome, namely MAR X-29 [22]. This fragment was multimerized to reach a comparable size to that of the MAR 1–68 core, resulting in a 660 bp sequence. The AT-rich elements from MAR 1–68 did not mediate any transcription increase whereas the multimerized X-29 AT core mediated a small but significant increase in transcription when compared to the control vector containing a spacer DNA of similar length (Fig. 3A). The AT core sequences from each MAR also behaved distinctly in terms of the anti-silencing effect; while the AT core sequences from MAR 1–68 did not show any anti-silencing effect, those from MAR X-29 contributed significant anti-silencing activity relative to their corresponding control vectors, with only 30% of the total polyclonal population of transfected cells being silenced. Overall, we concluded that the AT-rich portions of the MAR in isolation, that is in the absence of the binding site-containing flanking sequences, display little anti-silencing or transcription augmenting activities.

### Synergy of the AT-rich Core and Flanking Regions Mediates Full MAR Activity

The MAR 1–68 extended core, which includes parts of the flanking sequences (construct A of Fig. 1A), displayed significant MAR activity, while the minimal core composed only of AT-rich sequences did not (Fig. 3A). Thus, we next assessed whether a functional synergy may occur between the core region and different portions of the flanking regions. The 5′ and 3′ flanking regions (Fig. 1A constructs B and C, respectively) and their smaller subfragments (constructs D to G) were inserted between the extended AT core region and the SV40 promoter. Transfection assays indicated that the combination of the extended core region with either the full-length 5′ or 3′ flanking regions exerted a synergistic activation of GFP transgene expression, yielding a similar or greater effect than that mediated by the full length MAR, while either flanking region alone did not (Fig. 2 and 3B). Interestingly, the 3′ flanking region yielded the most potent combination, with close to half of the cells highly expressing GFP, although it did not exert any GFP up-regulation on its own. Consistently, this combination also mediated the lowest number of silent cells. This suggested that elements such as transcriptional factor binding sites present in this 3′ flanking region may not be sufficient for transcriptional activation, but that they may instead potentiate the function of the AT-rich core. None of the smaller fragments of MAR 1–68 flanking regions showed a synergistic effect with the AT core extended region, neither for GFP gene activation nor for the anti-silencing effects, implying that multiple DNA sequences and/or transcription factor binding sites might be needed.

Oligonucleotides containing various MAR 1–68 transcription factor binding motifs were thus multimerized in random combinations, in an attempt to mimic the MAR flanking sequences. When various combinations and numbers of the transcription factor binding motifs were added to the extended core, they had little influence overall on the occurrence of silent cells (Fig. S4A). Nevertheless, the extended core flanked by specific combinations of motifs yielded elevated numbers of highly expressing cells when compared to the full length MAR, whereas the mutimerized motifs alone had little or no effect on their own (Fig. S4B and data not shown). For instance, several combinations of the binding motifs for Hox, NMP4, CEBP, Gsh, SATB1 and Fast, as found on the MAR 3′ flanking region, significantly increased the effect of the extended core to increase expression, and the average number of high-expressor cells surpassed that obtained from the full length MAR. This indicated that multiple transcription factor binding motifs may be needed to mediate the transcriptional augmentation activity of the full length MAR. However, a more extensive and systematic study may be needed to fully understand the nature and organization of the binding motifs that mediate MAR activity.

Overall, we concluded that the extended core suffices to mediate full anti-silencing effects, as obtained from the full length MAR, while maximal transcriptional activation requires additional elements such as transcription factor binding motifs. Thus, distinct combinations of functional elements are required to reconstitute the two types of effects. A more extensive and systematic study of such combinations may be needed to fully understand the nature and organization of the binding motifs that mediate this activity.

### Distinct MAR Components Increase Transgene Copy Number and Expression

**Figure 4:**
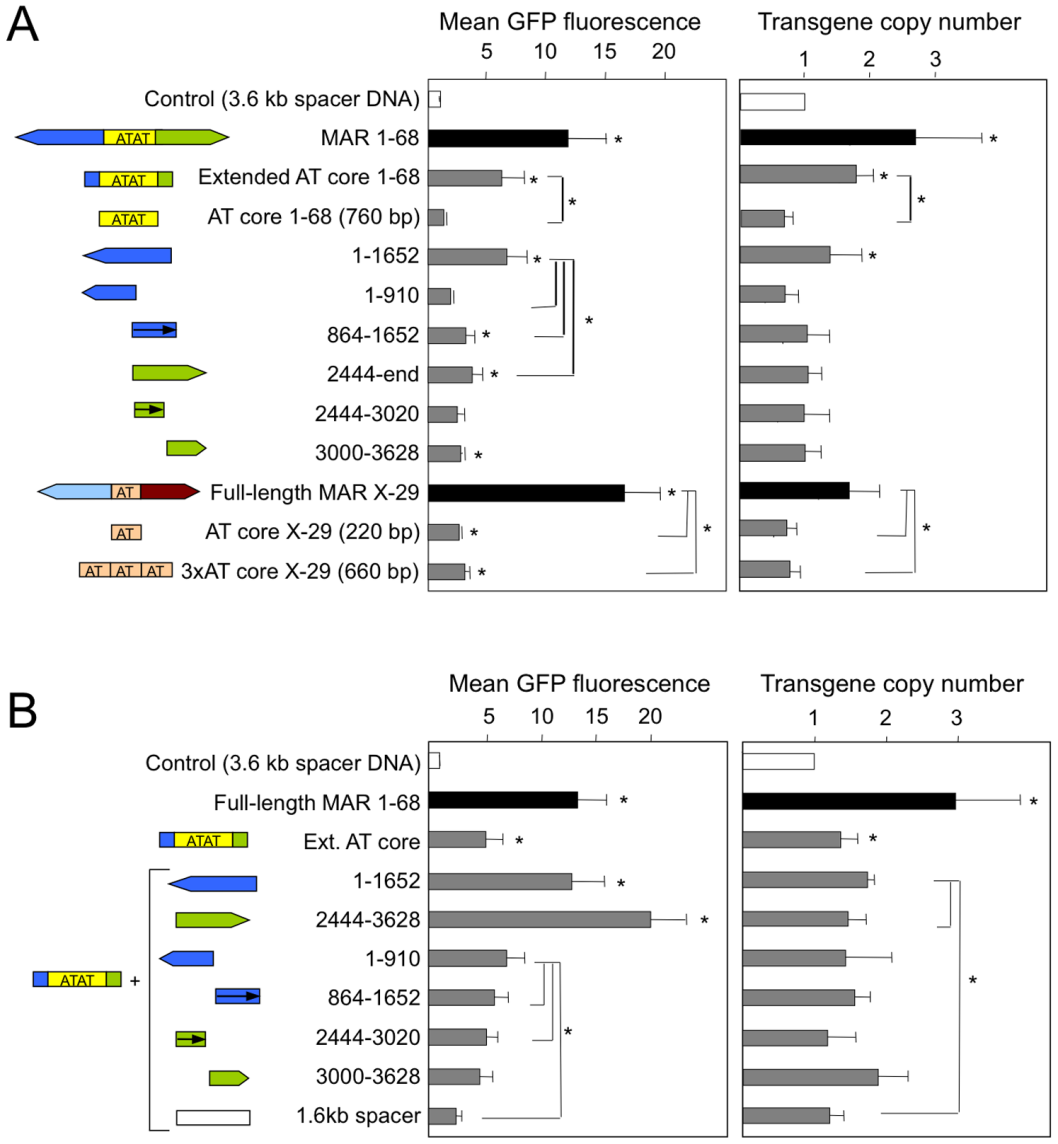
Effect of human MAR 1–68 and X-29 and derivatives on GFP expression and transgene copy number. (A) The mean GFP fluorescence and transgene copy numbers were determined from polyclonal cell pools generated using the illustrated constructs as described in the legend to Fig. 2. The relative GFP transgene copy number was determined by quantitative PCR using total genomic DNA isolated from transfected cell pools, and values were normalized to those of the GAPDH cellular gene. GFP expression levels and transgene copy numbers are expressed as the fold change relative to those obtained from control cells transfected with construct containing 3.6 kb of spacer DNA instead of the MAR, which was set to 1. Results represent the mean±SD of 3 independent experiments. Significant differences relative to the corresponding control construct containing spacer DNA of the same size, as illustrated in Suppl. Fig. S5, are indicated by stars above each bar, whereas line-associated stars indicate significant differences between the indicated constructs (Student test, P<0.05).

Previous studies have shown that MAR 1–68 mediates high gene expression in part by increasing the number of transgene copies that recombine with- and therefore stably integrate into- the cell genome, in addition to its transcriptional augmentation and antisilencing activities [20], [23]. To examine which MAR 1–68 constituents may specifically affect transgene copy number, we determined the average number of genome-integrated transgene copies by quantitative PCR, and correlated these values to the mean GFP fluorescence of total polyclonal CHO cell populations. Addition of the full length MAR 1–68 or MAR X-29 yielded a 12- and 17-fold increase of GFP fluorescence, respectively, when compared to the MAR-devoid control plasmid (Fig. 4A). MAR 1–68 significantly increased the number of integrated transgene copies as compared to the control vector, whereas MAR X-29 did not. When normalized to the transgene copy number, the MAR X-29 mediated a 10-fold increase of transgene expression, whereas a 4 to 5-fold increase was mediated by MAR 1–68. This indicated that the MAR 1–68 effect relies in part on a higher transgene copy number, whereas MAR X-29 mediates mainly transcriptional augmentation.

The extended AT core of MAR 1–68 showed an approximately 1.8-fold increase of the number of integrated GFP transgene copies, whereas the AT-rich core alone or the flanking sequences alone had lower or no effect (Fig. 4A and Fig. S5). This indicated that the ability of MAR 1–68 to increase transgene integration results mainly from the extended AT-rich core, but that it requires other sequences than the AT-rich repeats.

When normalized to the transgene copy number, the full-length flanking sequences displayed the highest increase in transcription, with a 4 to 5-fold effect that is comparable to that of the full-length MAR 1–68. However, this effect could not be ascribed to specific portions of the flanking sequences. This confirmed earlier indications that the flanking sequences activate gene transcription on their own, and that the combination of multiple sequence motifs may contribute to this effect. The MAR 1–68 extended core also mediated a 3–4 fold transcriptional augmentation effect, whereas its minimal core did not. Similarly, the minimal core of MAR X-29 did not increase copy number, and it mediated little transcriptional increase relative to the transgene copy number when compared to the full length MAR (2-fold vs. 17-fold, Fig. 4A and S5). This implied that the flanking sequences are required together with the core to obtain the full transcriptional augmentation effect.

This possibility was assessed by combining the MAR 1–68 flanking sequences with the extended core, which yielded GFP expression levels similar to that of the full-length MAR for the 5′ sequence, and even higher levels for the 3′ sequence (Fig. 4B). This finding correlates well with the prior finding that this combination yielded the highest proportion of highly expressing cells and the lowest silent cell counts. However, inclusion of the flanking sequences had little or no effect on transgene copy number (Fig. 4B), further indicating that distinct portions of the MAR mediate the transcriptional activation and copy number increase effects.

Overall, we therefore concluded that the transgene copy number-increasing effect requires multiple components of MAR 1–68, as present on the AT-rich core and adjacent flanking sequences. Although the extended core was found to mediate full antisilencing activity, it was unable alone to account for the full transcriptional augmentation activity of MAR 1–68 (Fig. 3A and 4A). Either one of the MAR 1–68 flanking sequences activates transcription in absence of the AT-rich core, and their combination with the antisilencing effect of the extended core was required for maximal transgene expression. Thus, the MAR 1–68 appears to be composed of a central AT-rich portion that mediates mostly the antisilencing and transgene integration effects, whereas transcriptional augmentation requires the flanking sequences.

### MAR A/T-rich Cores Display a Specific Chromatin Signature

**Figure 5:**
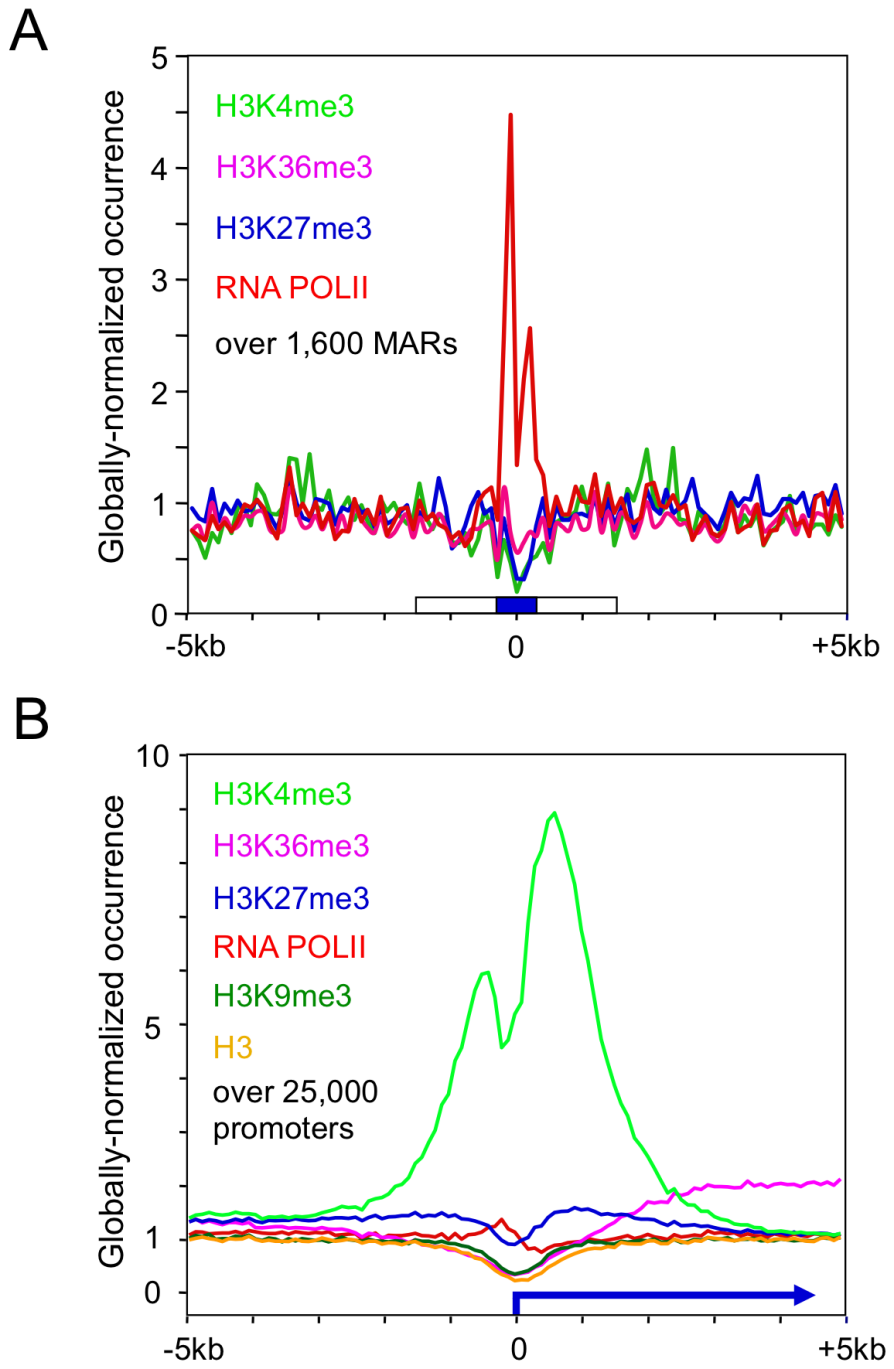
Association of human MARs with a specific chromatin pattern. A) 1683 predicted human MAR genomic locations were aligned using the central positions of their AT rich cores. ChiP-Seq profiles were calculated over the MAR collection for the histone modifications H3K4me3, H3K27me3, H3K36me3 and for RNA Polymerase II. (B) 25000 RefSeq promoters were aligned at their respective TSS positions and oriented according to the direction of transcription. ChiP-Seq profiles were calculated over the promoter collection for indicated histone modification, and for the RNA Pol II. Tag counts were normalized globally and they are expressed as a fold change over the non-precipitated input DNA profile.

The effect of transcription factors on effecting transcription initiation complex assembly and specific chromatin structure changes has been well documented [29]. For instance, the association of particular regulators such as CTCF and NF1 with the occurrence of chromatin domain boundaries has been associated to the antisilencing and insulator effects of these transcription factors [30]–[33]. However, the possible effect of the AT-rich sequences of MARs on chromatin structure remains uncharacterized. To avoid possible artefacts or biases linked to the study of a single or few genomic elements, this was assessed on approximately 1600 strong human MAR elements predicted using the SMARScan program, as this approach was shown to identify transcriptionally active MAR elements [22], and because MARs, such the human elements studied here, possess similar transcription increasing activities in cells types of various types and origins, including hamster, mouse and human cells [22], [23]. The MAR elements were aligned at the center of their AT-rich cores, and the occurrence of specific chromatin marks was determined using previously published datasets [34], [35]. Whereas an enrichment in particular histone marks was not observed over the MARs, a relative depletion of histones was noted over the AT core (Fig. 5A and Fig. S6). Interestingly, an enrichment of the CTCF insulator protein but not of another transcription factor, STAT1, was also observed over the nucleosome-depleted core sequences (Fig. S6 and data not shown).

A depletion of nucleosomes has been previously reported to occur over active promoters [29]. Thus, we next assessed whether RNA polymerase II might be enriched over the MARs. Strikingly, a prominent occurrence of RNA polymerase II was observed over the AT-rich domains, whereas the trimethylation of lysine 36 of histone H3 (H3K36me3), a marker of transcribed DNA, was not observed (Fig. 5A). When a similar analysis was performed over approximately 25000 promoters oriented relative to transcription initiation, a relative histone depletion was also observed over the transcriptional initiation sequences, whereas a gradual increase in the H3K36me3 mark was observed over the transcribed DNA, in agreement with previous studies (Fig. 5B) [29], [34], [35]. An RNA polymerase II peak was also observed over the promoter, but it was less prominent than the one observed over the MARs. A small peak is expected from active promoter sequences, as the transient association of the polymerase to the promoter is followed by its departure to transcribe downstream sequences [36]. Another histone mark specific of promoter sequences, H3K4me3, featured prominently around the promoter, whereas it was depleted over the MAR sequences. Overall, this indicated that the A/T-rich core of MARs are associated with a novel type of chromatin landscape that is nucleosome-depleted but RNA-polymerase II- and CTCF-enriched, and which is distinct from that found over active promoters.

## Discussion

Since their discovery, matrix attachment regions have been studied extensively to understand their proposed role in chromatin organization, gene expression and DNA replication. Since the proposal that they may mediate chromatin loop formation, and thereby act as chromatin boundary elements, MAR elements has been assessed for their ability to improve gene expression, for instance in protein production and for gene or cell therapy. This was often met with success, given their anti-silencing and transcriptional augmentation activities [14]. These features are not necessarily elicited by all MARs, however, and attempts to identify a consensus sequence or a molecular mode of action were met with limited success (e.g. [37]). In this study, we assessed the hypothesis that MARs may rely on both an AT-rich core and on adjacent binding sites for specific transcription factor, as suggested by earlier bioinformatics modelling studies [22]. Here, we report that the three functions ascribed to MAR elements, namely the anti-silencing, transcriptional and transgene integration augmenting activities, rely at least in part on a combination of these two types of DNA elements.

AT-rich core sequences, such as those of the MAR 1–68 and X-29, consist of essentially uninterrupted rows of alternating A and T bases, which were previously associated with DNA curvature and facilitated base pair unwinding. These DNA curvature and base pair unwinding properties were proposed to be key features for MAR activity in terms of increased transgene expression [24]. Some of these AT cores may act as binding sites for specific transcription factors that target AT-rich sequences, such as SATB1 and SAF-A [10], [11], [14], [24], [38]. Here, we found that the extended core region of MAR 1–68 is unable to provide a high transcriptional augmentation effect, whereas it mediates the full anti-silencing activity of the MAR. Interestingly, the mere presence of AT dinucleotide repeats of either MAR 1–68 or MAR X-29 does not suffice to mediate the anti-silencing effect. This implies that the anti-silencing activity requires as yet unidentified sequences immediately flanking the core in addition to the AT-dinucleotide repeats.

Extension of the MAR 1–68 core to contain adjacent flanking sequences restored the transcriptional augmentation activity to levels equal or higher than those obtained with the full length MAR. When tested alone, the flanking sequences also displayed significant transcriptional augmentation activities. However, when further molecular dissection of the determinants of transcriptional augmentation was performed, the loss or lack of activity of smaller derivatives indicated that a single short sequence or transcription binding motif could not fully account for this activity. Consistently, combination of the core with multiple transcription factor binding motifs, as found on the flanking sequence, was required to restore the full transcriptional augmentation activity. Interestingly, particular combinations of binding motifs for the SatB1, Hox, Gsh and CEBP proteins mediated even higher expression levels than the natural MAR element. Overall, we thus conclude that the AT-rich core mainly mediates an anti-silencing effect, whereas the transcriptional augmentation effect results more prominently from the transcription factor binding motifs present on the flanking sequences. Nevertheless, this distinction is not absolute, as the flanking sequences also mediate some anti-silencing activity, whereas the extended core has a reduced but still significant transcriptional augmentation activity. This may be explained by the occurrence of transcription factor binding sites adjacent to the AT-rich minimal core, whereas some AT-rich patches also occur within the flanking sequences.

The action of transcription factors on chromatin structure and the resulting activation of transcription has been well documented in the context of promoters and enhancers [29]. However, how the AT-rich core may contribute to gene expression and whether it may act to regulate chromatin structure has remained mainly untested. Analysis of chromatin features around MARs did not reveal an enrichment in particular histone marks. Rather, a general decrease in histones was noted over the AT-rich cores, as observed previously over promoters. The lack of nucleosome over the core can be well explained by the previous demonstration that stretches of As and Ts that extend over 10 base pairs, as found in MAR cores, act to exclude DNA wrapping around the nucleosome in vitro and in vivo [39]. Surprisingly, we rather observed a high enrichment for RNA polymerase II over the cores, but this was not associated with the histone marks characteristic of transcribed DNA, and the chromatin pattern was distinct from that of active promoters. These observations taken together shed a new light on the molecular modes of action of MAR elements.

The lack of histone over the AT-rich core may readily explain the chromatin domain boundary and antisilencing activities of MARs. The propagation of heterochromatin along the DNA is associated to the binding of heterochromatin-specific proteins such as HP1α with nucleosomes bearing silent chromatin marks. This, in turn, allows the recruitment of histone-modifying proteins such as histone methyl transferases that act to deposit additional silencing marks onto adjacent nucleosomes, and so on, to mediate the autopropagation of the silencing chromatin structure along the chromosomal DNA [40]. The interruption of such nucleosomal arrays by the depletion of nucleosomes over MARs and promoters has been proposed to block the propagation of heterochromatin along the DNA, and thereby to prevent the silencing of adjacent genes [41]. Thus, the lack of nucleosomes over the AT rich core of MAR elements and the association with the CTCF insulator protein should act as a road-block to the propagation of heterochromatin over adjacent transgenes, thereby mediating the MAR antisilencing activity.

Another feature of MARs is to augment the transcription of adjacent genes [20]. The association of MAR cores with RNA polymerase II is expected to provide a high local concentration of the polymerase in their neighbourhood. Indeed, MAR-binding proteins such as SAT B1 and CTCF have been associated with the formation of DNA loops that may bring these epigenetic regulatory sequences in proximity to enhancer and promoter sequences driving the expression of their target genes [4]–[6]. Thereby, the MAR elements may bring the nuclear matrix, consisting mainly of transcription factors and RNA processing enzymes [21], in the proximity of transcriptional initiation sites at promoters. This may in turn facilitate the loading of the polymerase over adjacent promoters, as also facilitated by transcription factors. This finding may readily explain the continuous transcription previously observed when a MAR is positioned next to a transgene, whereas, in the absence of the MAR, transcription occurred in bursts separated by transcriptionally inactive periods [20]. Overall, these observations and the mechanistic model they imply may provide a unifying interpretation of the role of both MAR elements and promoters to mediate chromatin domain boundaries as well as of their cooperative action on gene expression [30]. They also provide an explanation for previous failures to associate MAR activities with specific consensus sequences or simple sequence motifs, as the multiple MAR actions appear to result from the combinations of various types of functional DNA elements.

## Materials and Methods

### Plasmids and Constructs

The human MAR 1–68 and X-29 were previously identified from a screen of the human genome using the SMARScan I MAR predicting software [22]. MAR 1–68 and X-29 of 3.6 and 2.9 kb in length, respectively, were cloned into the pEGFP control expression vector upstream of the SV40 early promoter that drives the green fluorescent protein reporter gene expression.

The 5′ (1–1652, 1–910 & 864–1652) and the 3′ (2444–3628, 2444–3020 & 3000 to 3628) flanking regions of the MAR 1–68 were amplified using primers listed in Supplementary Table S1. Bidirectional cloning was achieved by digesting the PCR amplified fragments with BamH1 and subsequently inserting them in pEGFP plasmid digested with BglII. The AT-rich core region of MAR 1–68 (770 bp) was removed from MAR 1–68 by digestion with PfIFI and MfEI. The PfIFI was filled-in with Klenow (Klenow fragment, NEB) and the fragment was ligated to pEGFP plasmid at the multiple cloning sites, digested with EcoRI (compatible with MfEI) and EcoRV.

The AT-rich core region of another human MAR, namely MAR X-29, was amplified using primers listed in Supplementary Table S1. The PCR amplified fragment was digested with EcoRV and ligated into EcoRV digested pEGFP plasmid. Alternatively, the 220 bp PCR amplified product of the AT-rich core region of MAR X-29 was digested completely by EcoRV and purified using the Wizard® SV and PCR clean-up System (Promega), as recommended by the manufacturer, followed by self ligation, giving rise to a series of DNA fragments with increasing copy numbers in various orientations. Self-ligated products were separated by gel electrophoresis and fragments in the range from 400 bp to 1 kb were purified using Wizard® SV and PCR clean-up System (Promega) and cloned into pEGFP plasmid. A clone containing the largest insert of 660 bps in length was selected and sequenced.

As negative controls, the MAR fragments were replaced by DNA sequences that encode a part of the Utrophin and/or luciferase genes. The control DNA fragments of 240 to 1652 bp in length were generated by PCR, using luciferase cDNA as template and primers listed in Supplementary Table S2. The control DNA for the full-length MARs of 3.6 kb consists of a 2 kb fragment from Utrophin plus a 1.6 kb fragment from luciferase. The Utrophin fragment was generated by PCR using the plasmid CMV–Utrophin [42] as template and the pair of primers listed in Supplementary Table S2. The 1.6 kb fragment derived from the luciferase cDNA was inserted between the utrophin 2 kb fragment and the SV40 promoter. All control DNA fragments were cloned into pEGFP plasmid. All plasmids, DNA vectors, and other renewable resources, as generated in this study, will be made freely available for non-profit research use, unless specifically restricted by some other party.

### Generation of Multimerized Putative Binding Sites of Transcription Factors (TFs)

Oligonucleotides containing putative binding sites of TFs were designed to avoid the formation of inverted repeats after oligomerization by ligation and cleavage by BamHI and BglII (see Suppl. Table S3). After annealing of the sense and antisense strands, oligonucleotides were 5′ phosphorylated and self-ligated. Oligomers above 100 bp in length were purified as described above and inserted into the pEGFP vector containing the MAR 1–68 AT-rich core region (1429–2880). Individual clones were characterized by sequencing.

### Cell Culture and Stable Transfection

The CHO DG44 cell line [43] was cultivated in DMEM:F12 (Gibco-Invitrogen) supplemented with 10% FBS (Gibco-Invitrogen) and hypoxanthine and thymidine (HT, Gibco-Invitrogen). CHO DG44 cells were seeded in 24-well plates at 900,000 cells per well and allowed to attach overnight. The following day, CHO cells were co-transfected with a pEGFP reporter construct containing various fragments from MAR 1–68, MAR X-29 or spacer DNA, and with pSV2neo (CLONTECH Laboratories, Inc.) in a molar ratio of 10∶1, using Roche FuGENE® 6 as the transfection reagent, as recommended by the manufacturer. The total amount of DNA was adjusted to 860 ng/15.6 mm-well, by the addition of pUC19 control DNA in order to maintain constant DNA concentrations in the transfection mixes. All plasmids were linearized with PvuI before transfection. 24 hours post-transfection, the cells were washed with PBS, treated with trypsin and transferred to T-75 cell-culture flasks containing DMEM medium supplemented with 10% FCS, 1X HT and 500 µg/ml geneticin. The use of selective medium was continued for 2–3 weeks, with frequent changes of medium to remove dead cells and debris.

### Cytofluorometry and Fluorescence Activated Cell (FACS) Sorting

At the end of the selection period, the polyclonal cell populations were harvested by trypsin-EDTA and resuspended in serum free synthetic ProCHO5 medium (Cambrex) for GFP fluorescence assays. GFP cytofluorometric analyses were acquired on a FACScalibur flow cytometer (Becton and Dickinson). 100,000 events were analyzed using the GFP (FL-1) channel with setting at 240 V and acquired data were analyzed using the WinMDI 2.8 software. Selected cells were sorted by flow cytometry using the FACSAriaII cell sorter (Becton and Dickinson) in order to obtain 4 sub-populations according to the following criteria: no, low, medium or very high GFP expression. After sorting, 1 million cells of each sub-population were expanded in T-25 tissue culture flasks containing DMEM-F12 complete medium with 500 µg/ml geneticin until confluency.

### Quantitative Analysis of the GFP Transgene Copy Number

DNA was isolated from FACS-sorted cells using the DNeasy Blood & Tissue Kit (Qiagen) following the manufacturer’s instructions. 6 ng of genomic DNA was analyzed by quantitative PCR to determine the copy number of the GFP transgene integrated in the genome of the CHO cells. Analyses were performed in the LightCycler® 480 (Roche), using the Light Cycler SYBR Green Master MIX (2X) and 0.4 µM (final concentration) of forward and reverse primers, in a final volume of 15 µl. The GFP and GAPDH primers sets used for quantitative DNA PCR amplifications are shown in Supplementary Table S4. The GFP reporter gene copy number was normalized and calculated relative to that of the GAPDH as described by [44]. Relative gene numbers were calculated using the equation E_B_^CtB^/E_A_^CtA,^ whereby E corresponds to the PCR efficiency estimated by the LinReg Method and CtA and CtB represent the cycle thresholds for the gene of interest (GFP) or the endogenous reference gene (GAPDH), respectively. The data are representative of three to four independent transfection experiments, each performed in triplicate.

### GFP mRNA Level Analysis

Total RNA was prepared from approximately 5×10^6^ transfected cells using the RNAeasy Kit (QIAGEN) following the manufacturer’s instructions. 1 µg of total RNA was reverse-transcribed with oligo (dT) primers, using First Strand cDNA Synthesis Kit (GE Healthcare) according to the manufacturer’s instructions. Quantitative PCR was performed with primers specific to GFP and eukaryotic elongation factor 1 α1 (eEFIA1) genes, on a Light Cycler 480 system (Roche) using Roche LightCycler® 480 SYBR Green Master MIX. The GFP and eEFIA primers used for quantitative PCR amplification are shown in Supplementary Table S4. To normalize for variation in RNA content, we used EEFIA1 as the endogenous reference gene. Relative GFP expression was quantified by qPCR as described above.

### Global ChiP-Seq Analysis of MAR Elements

Positions of the MAR elements within the human genome were determined using the SMARscan I software [22]. A total of 1697 MAR elements were found using a SMARscan score above 402 in the hs18 human genome version (March 2006). After remapping to the human genome version hg19 (Feb 2009) a total of 1683 MARs were retained. To analyze the ChIP-Seq profiles of MARs for histones, RNAPol II and transcription factors, we used published ChIP-Seq data sets from human CD4+ T cells [35] . Correlation analyses of ChIP-Seq tags and MAR predicted locations were performed using the ChIP-Cor tool available on the ChIP-Seq Analysis Server of the Swiss Institute of Bioinformatics (URL: http://ccg.vital-it.ch/chipseq/chip_cor.php). Cumulative ChIP-Seq tag counts were determined over the collection of 1683 human MARs for the genomic regions +/−5 kb from the central positions of the AT rich core of MARs. Tags were counted in consecutive windows of 100 bp and the fold change over the genome-wide average tag count was calculated for each window position. As the MAR AT-rich cores have low complexity sequences with generally lower tag counts, further normalization was required using non-precipitated data set. Normalization over the non-precipitated set was calculated as a fold change for each window position over corrected tag count for the non-precipitated MAR ChIP-Seq profile. Correction was taking into account the global number of tags obtained in the two experiments (tag count of non-precipitated MAR profile multiplied by factor K = total tags X/total tags non-precipitated MAR profile). ChIP-Seq profiles were obtained for the following histone variants and histone modifications: H2AZ variant, H3K4me1, H3K4me2, H3K4me3, H3K27me1, H3K27me2, H3K27me3, H3K36me1 and H3K36me3. Profiles were in addition obtained for the RNAPol II, and for transcription factors: CTCF and STAT1 (stimulated and non-stimulated cells). Similar bioinformatics tools from the ChIP-Seq Analysis Server of the Swiss Institute of Bioinformatics and normalization method were used to correlate ∼25000 RefSeq transcriptional start sites with histone modifications.

## Supporting Information

Figure S1 **GFP transgene expression is related to the transgene copy number.** CHO-DG44 were stably co-transfected with the constructs containing the full-length MAR 1–68 or the spacer control DNA and with a plasmid encoding an antibiotic resistance gene. The polyclonal cell pools obtained after 1 month of antibiotic selection post-transfection were subjected to cytofluorometry analysis and cell sorting. The cells were sorted into 4 populations according to the levels of GFP fluorescence, as illustrated in panel (A), and they were subsequently expanded for analysis of the relative GFP transgene copy number, illustrated as described in the legend to Fig. 4 (B). Significant differences are indicated by star signs (Student test, P<0.05).
(PDF)

Figure S2 **GFP mRNA levels are related to the transgene copy number.** mRNA was isolated from the cells of Suppl. Fig. S1, stably transfected with the GFP expression plasmid containing or not MAR 1–68 and sorted on the basis of their high GFP fluorescence or lack thereof. The same amount of total mRNA was used for reverse transcription and quantitative PCR assays to determine the relative fold change of GFP transcript levels. Experimental values were normalized to that of the GAPDH mRNA and they are expressed as the fold change relative to that of the control cells transfected with the MAR devoid construct, which was set to 1. Some of the significant differences are indicated by star signs (Student test, P<0.05).
(PDF)

Figure S3 **Effect of negative control DNA sequences on the occurence of silent and high expressor cells.** Spacer DNA of various lengths (3.6 kb to 200 bp), consisting of part of the utrophin or luciferase coding sequences, were used to replace the full-length MAR 1–68 or its deletions derivatives. The proportion of silent and high-expressor cells were determined and displayed as described in the legend to Fig. 2.
(PDF)

Figure S4 **Effect of transcription factor DNA binding motifs on the occurence of silent and high expressor cells.** Oligonucleotides corresponding to DNA sequence motifs predicted to act as binding sites for the SATB1, Hox, Gsh, Fast-1 and CEBP transcription factors by the MatInspector software were mixed and inserted randomly downstream of the extended AT-rich core and upstream of the SV40 promoter of the GFP expression vector depicted in Fig. 1A. The number and order of the binding motifs were determined by DNA sequencing, as indicated, and various combinations containing from 2 to 9 motifs were randomly selected for analysis. The proportion of silent and high-expressor cells were determined and displayed as described in the legend to Fig. 2. Significant differences relative to the construct containing the extended AT core alone are indicated by stars above each bar, whereas line-associated stars indicate significant differences between the indicated constructs (Student test, P<0.05).
(PDF)

Figure S5 **Effect of negative control DNA sequences on the average GFP fluorescence and transgene copy number.** Spacer DNA of various lengths (3.6 kb to 200 bp), consisting of part of the utrophin or luciferase coding sequences, were used to replace the full-length MAR 1–68 or its derivatives. The average GFP fluorescence and transgene copy numbers were determined from polyclonal cell pools generated using the illustrated constructs as described in the legend to Fig. 4.
(PDF)

Figure S6 **1683 predicted human MAR genomic locations were aligned using the central positions of their AT rich cores.** ChiP-Seq profiles were calculated over the MAR collection for association with the CTCF transcription factor, for DNAse hypersensitive sites and for the H2AZ histone variant. Tag counts were normalized globally and they are expressed as a fold change over the non-precipitated input DNA profile.
(PDF)

Table S1 **Primer sets used to amplify portions of MAR 1–68 and MAR X-29.**
(PDF)

Table S2 **Primers sets used to amplify control cDNA sequences.**
(PDF)

Table S3 **Oligonucleotides containing transcription factor binding motifs.**
(PDF)

Table S4 **Quantitative PCR primer sets for GFP, GAPDH and eEFIA.**
(PDF)
