# Supplementary material for: Molecular Characterization of a Human Matrix Attachment Region Epigenetic Regulator
Source: PLoS One. 2013 Nov 14;8(11):e79262. doi: 10.1371/journal.pone.0079262 (PMC3828356; doi:10.1371/journal.pone.0079262)
Supplement: Figure S1 — GFP transgene expression is related to the transgene copy number. CHO-DG44 were stably co-transfected with the constructs containing the full-length MAR 1–68 or the spacer control DNA and with a plasmid encoding an antibiotic resistance gene. The polyclonal cell pools obtained after 1 month of antibiotic selection post-transfection were subjected to cytofluorometry analysis and cell sorting. The cells were sorted into 4 populations according to the levels of GFP fluorescence, as illustrated in panel (A), and they were subsequently expanded for analysis of the relative GFP transgene copy number, illustrated as described in the legend to Fig. 4 (B). Significant differences are indicated by star signs (Student test, P<0.05). (PDF) [file pone.0079262.s001.pdf]

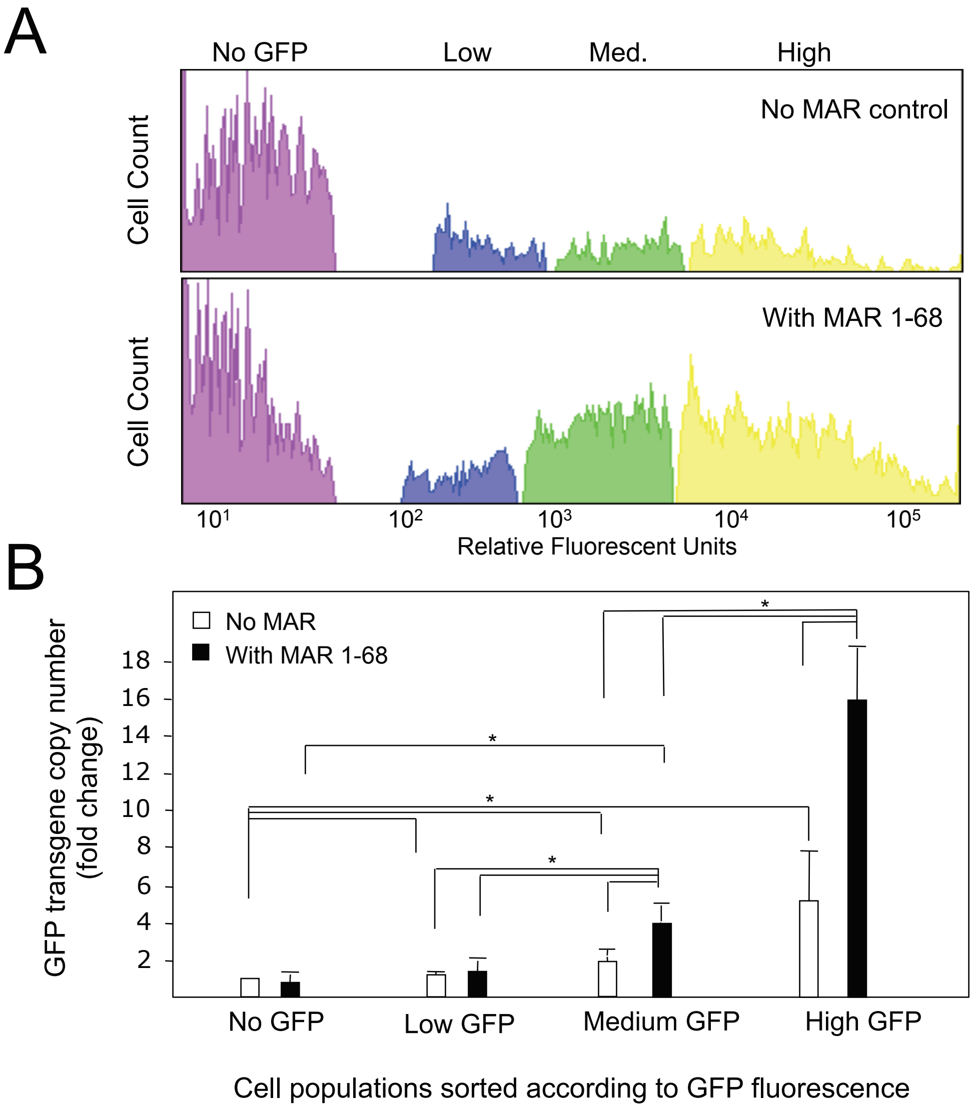

Figure S1. GFP transgene expression is related to the transgene copy number. CHO-DG44 were stably co-transfected with the constructs containing the full-length MAR 1-68 or the spacer control DNA and with a plasmid encoding an antibiotic resistance gene. The polyclonal cell pools obtained after 1 month of antibiotic selection post-transfection were subjected to cytofluorometry analysis and cell sorting. The cells were sorted into 4 populations according to the levels of GFP fluorescence, as illustrated in panel (A), and they were subsequently expanded for analysis of the relative GFP transgene copy number, illustrated as described in the legend to Fig. 4 (B). Significant differences are indicated by star signs (Student test, $P<0.05$).
