# Supplementary material for: Molecular Characterization of a Human Matrix Attachment Region Epigenetic Regulator
Source: PLoS One. 2013 Nov 14;8(11):e79262. doi: 10.1371/journal.pone.0079262 (PMC3828356; doi:10.1371/journal.pone.0079262)
Supplement: Figure S2 — GFP mRNA levels are related to the transgene copy number. mRNA was isolated from the cells of Suppl. Fig. S1, stably transfected with the GFP expression plasmid containing or not MAR 1–68 and sorted on the basis of their high GFP fluorescence or lack thereof. The same amount of total mRNA was used for reverse transcription and quantitative PCR assays to determine the relative fold change of GFP transcript levels. Experimental values were normalized to that of the GAPDH mRNA and they are expressed as the fold change relative to that of the control cells transfected with the MAR devoid construct, which was set to 1. Some of the significant differences are indicated by star signs (Student test, P<0.05). (PDF) [file pone.0079262.s002.pdf]

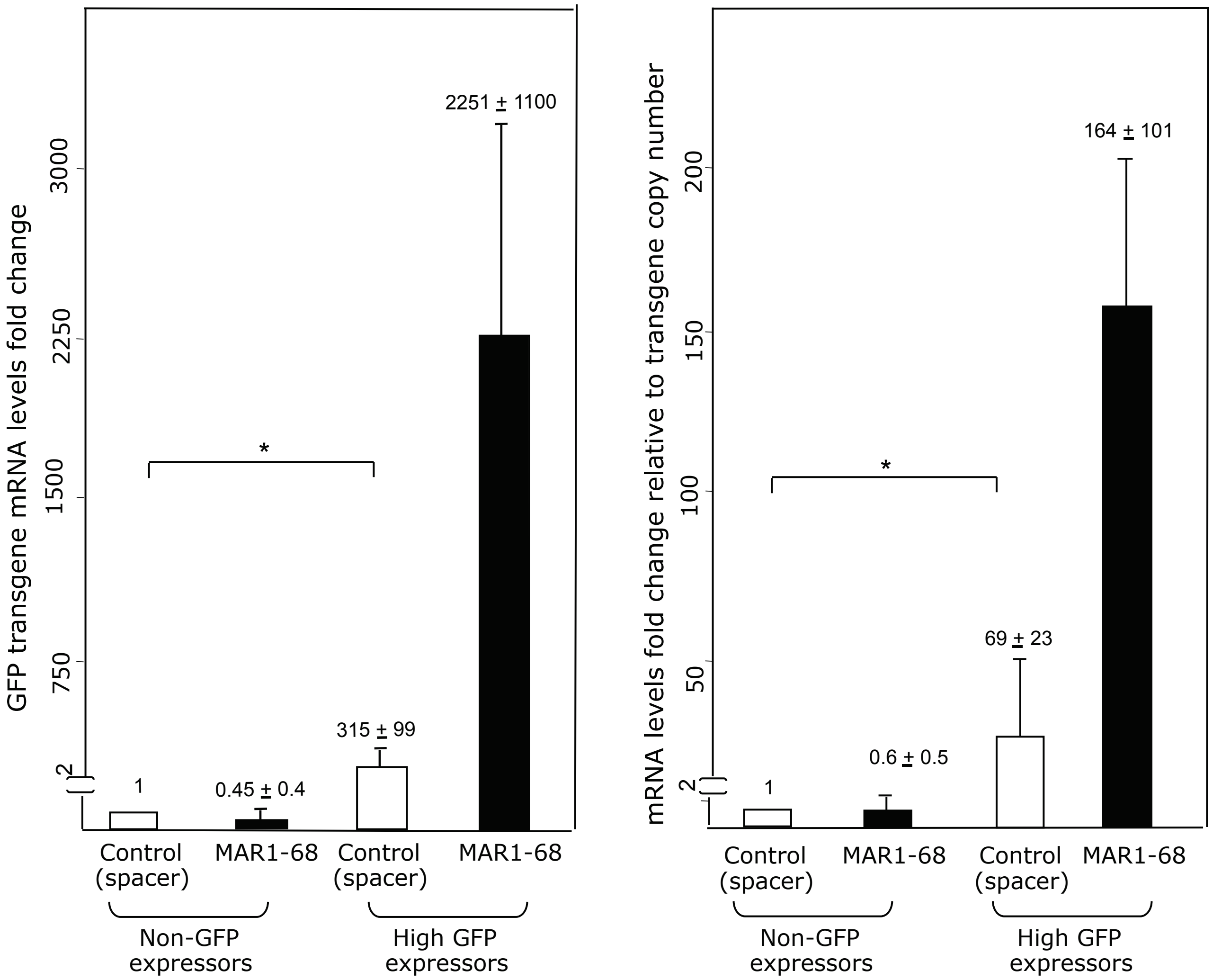

Figure S2. GFP mRNA levels are related to the transgene copy number. mRNA was isolated from the cells of Suppl. Fig. S1, stably transfected with the GFP expression plasmid containing or not MAR 1-68 and sorted on the basis of their high GFP fluorescence or lack thereof. The same amount of total mRNA was used for reverse transcription and quantitative PCR assays to determine the relative fold change of GFP transcript levels. Experimental values were normalized to that of the GAPDH mRNA and they are expressed as the fold change relative to that of the control cells transfected with the MAR devoid construct, which was set to 1. Some of the significant differences are indicated by star signs (Student test, $P<0.05$).
