# Supplementary material for: Molecular Characterization of a Human Matrix Attachment Region Epigenetic Regulator
Source: PLoS One. 2013 Nov 14;8(11):e79262. doi: 10.1371/journal.pone.0079262 (PMC3828356; doi:10.1371/journal.pone.0079262)
Supplement: Figure S3 — Effect of negative control DNA sequences on the occurence of silent and high expressor cells. Spacer DNA of various lengths (3.6 kb to 200 bp), consisting of part of the utrophin or luciferase coding sequences, were used to replace the full-length MAR 1–68 or its deletions derivatives. The proportion of silent and high-expressor cells were determined and displayed as described in the legend to Fig. 2. (PDF) [file pone.0079262.s003.pdf]

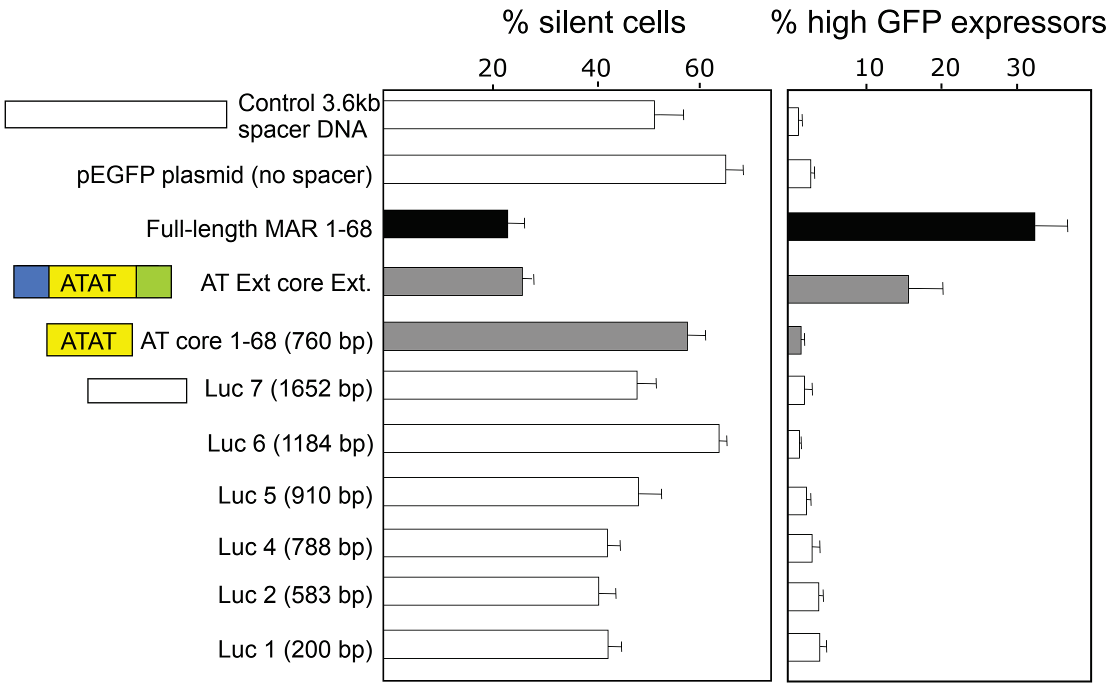

Figure S3. Effect of negative control DNA sequences on the occurence of silent and high expressor cells. Spacer DNA of various lengths (3.6 kb to 200 bp), consisting of part of the utrophin or luciferase coding sequences, were used to replace the full-length MAR 1-68 or its deletions derivatives. The proportion of silent and high-expressor cells were determined and displayed as described in the legend to Fig. 2.
