# Supplementary material for: Molecular Characterization of a Human Matrix Attachment Region Epigenetic Regulator
Source: PLoS One. 2013 Nov 14;8(11):e79262. doi: 10.1371/journal.pone.0079262 (PMC3828356; doi:10.1371/journal.pone.0079262)
Supplement: Figure S4 — Effect of transcription factor DNA binding motifs on the occurence of silent and high expressor cells. Oligonucleotides corresponding to DNA sequence motifs predicted to act as binding sites for the SATB1, Hox, Gsh, Fast-1 and CEBP transcription factors by the MatInspector software were mixed and inserted randomly downstream of the extended AT-rich core and upstream of the SV40 promoter of the GFP expression vector depicted in Fig. 1A. The number and order of the binding motifs were determined by DNA sequencing, as indicated, and various combinations containing from 2 to 9 motifs were randomly selected for analysis. The proportion of silent and high-expressor cells were determined and displayed as described in the legend to Fig. 2. Significant differences relative to the construct containing the extended AT core alone are indicated by stars above each bar, whereas line-associated stars indicate significant differences between the indicated constructs (Student test, P<0.05). (PDF) [file pone.0079262.s004.pdf]

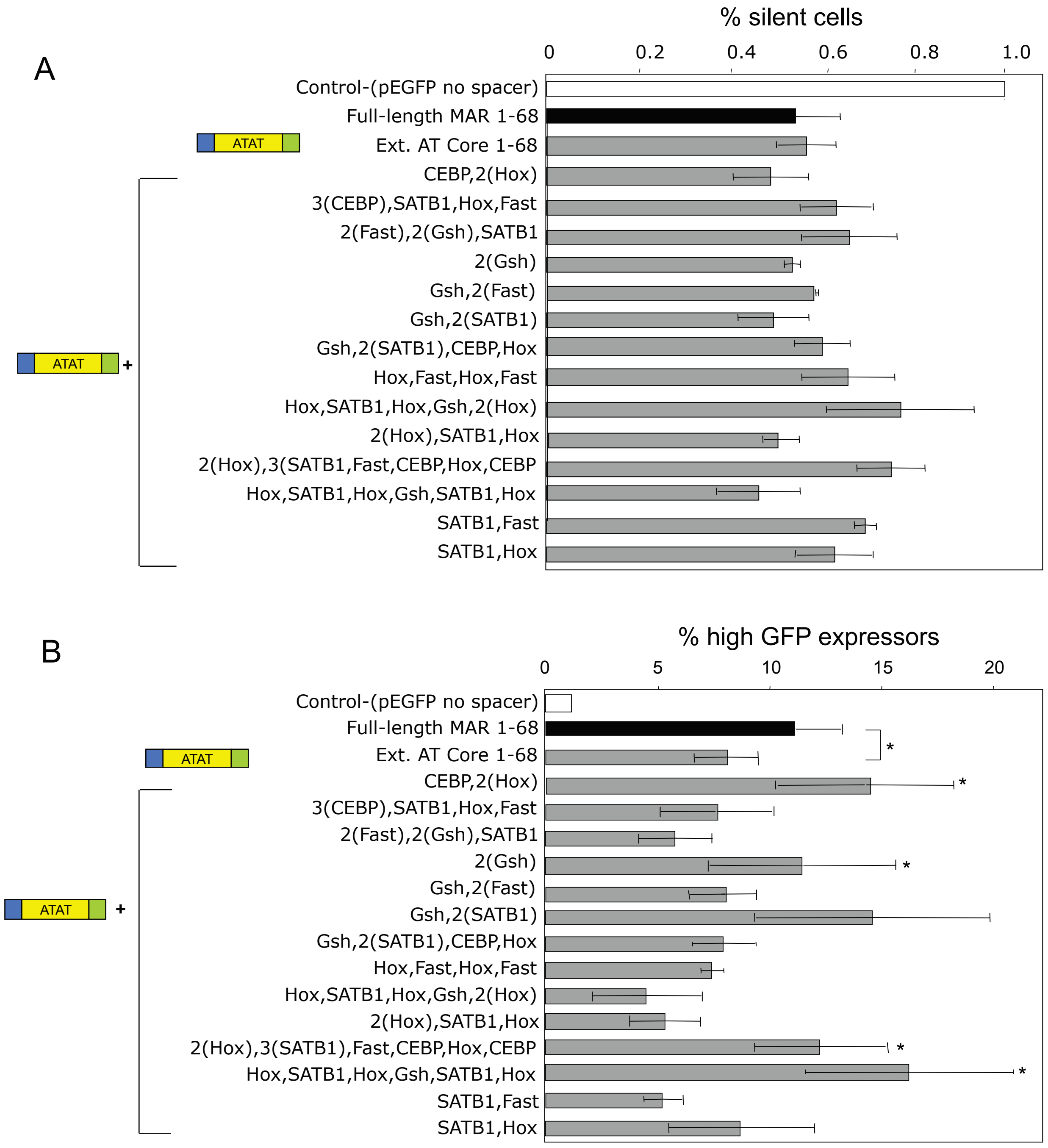

Figure S4. Effect of transcription factor DNA binding motifs on the occurence of silent and high expressor cells. Oligonucleotides corresponding to DNA sequence motifs predicted to act as binding sites for the SATB1, Hox, Gsh, Fast-1 and CEBP transcription factors by the MatInspector software were mixed and inserted randomly downstream of the extended AT-rich core and upstream of the SV40 promoter of the GFP expression vector depicted in Fig. 1A. The number and order of the binding motifs were determined by DNA sequencing, as indicated, and various combinations containing from 2 to 9 motifs were randomly selected for analysis. The proportion of silent and high-expressor cells were determined and displayed as described in the legend to Fig. 2. Significant differences relative to the construct containing the extended AT core alone are indicated by stars above each bar, whereas line-associated stars indicate significant differences between the indicated constructs (Student test, P<0.05).
