# Supplementary material for: Molecular Characterization of a Human Matrix Attachment Region Epigenetic Regulator
Source: PLoS One. 2013 Nov 14;8(11):e79262. doi: 10.1371/journal.pone.0079262 (PMC3828356; doi:10.1371/journal.pone.0079262)
Supplement: Figure S5 — Effect of negative control DNA sequences on the average GFP fluorescence and transgene copy number. Spacer DNA of various lengths (3.6 kb to 200 bp), consisting of part of the utrophin or luciferase coding sequences, were used to replace the full-length MAR 1–68 or its derivatives. The average GFP fluorescence and transgene copy numbers were determined from polyclonal cell pools generated using the illustrated constructs as described in the legend to Fig. 4. (PDF) [file pone.0079262.s005.pdf]

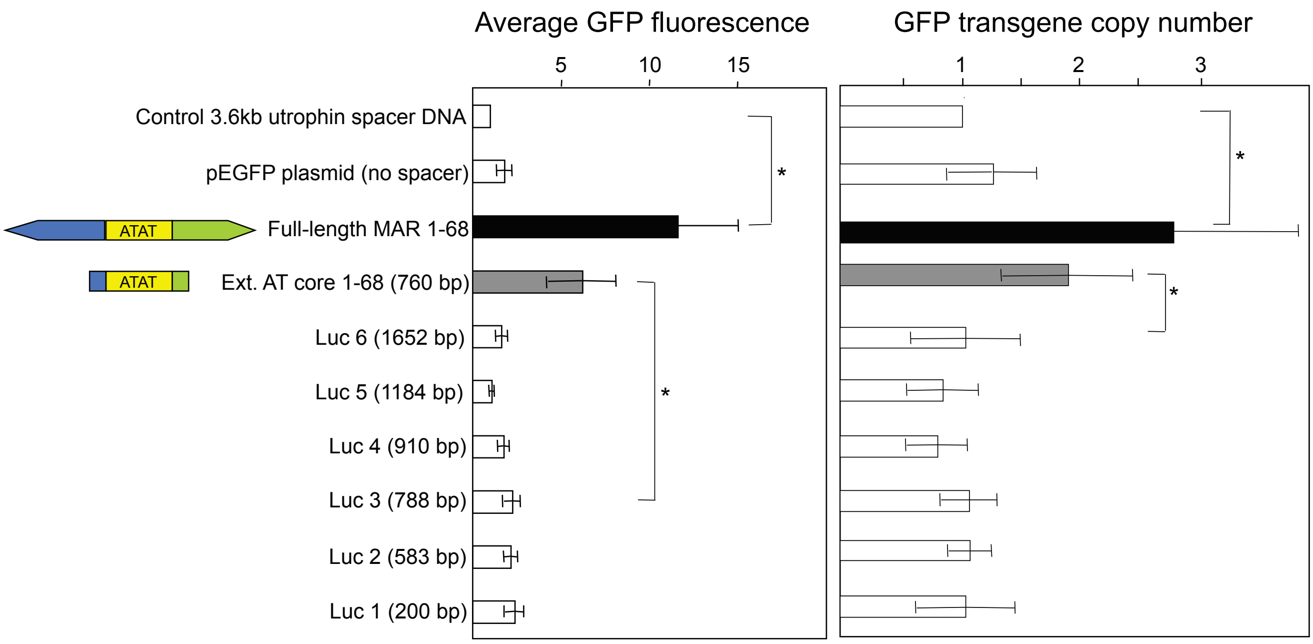

Figure S5. Effect of negative control DNA sequences on the average GFP fluorescence and transgene copy number. Spacer DNA of various lengths (3.6 kb to 200 bp), consisting of part of the utrophin or luciferase coding sequences, were used to replace the full-length MAR 1-68 or its deletions derivatives. The average GFP fluorescence and transgene copy numbers were determined from polyclonal cell pools generated using the illustrated constructs as described in the legend to Fig. 4.
