# Supplementary material for: Molecular Characterization of a Human Matrix Attachment Region Epigenetic Regulator
Source: PLoS One. 2013 Nov 14;8(11):e79262. doi: 10.1371/journal.pone.0079262 (PMC3828356; doi:10.1371/journal.pone.0079262)
Supplement: Figure S6 — 1683 predicted human MAR genomic locations were aligned using the central positions of their AT rich cores. ChiP-Seq profiles were calculated over the MAR collection for association with the CTCF transcription factor, for DNAse hypersensitive sites and for the H2AZ histone variant. Tag counts were normalized globally and they are expressed as a fold change over the non-precipitated input DNA profile. (PDF) [file pone.0079262.s006.pdf]

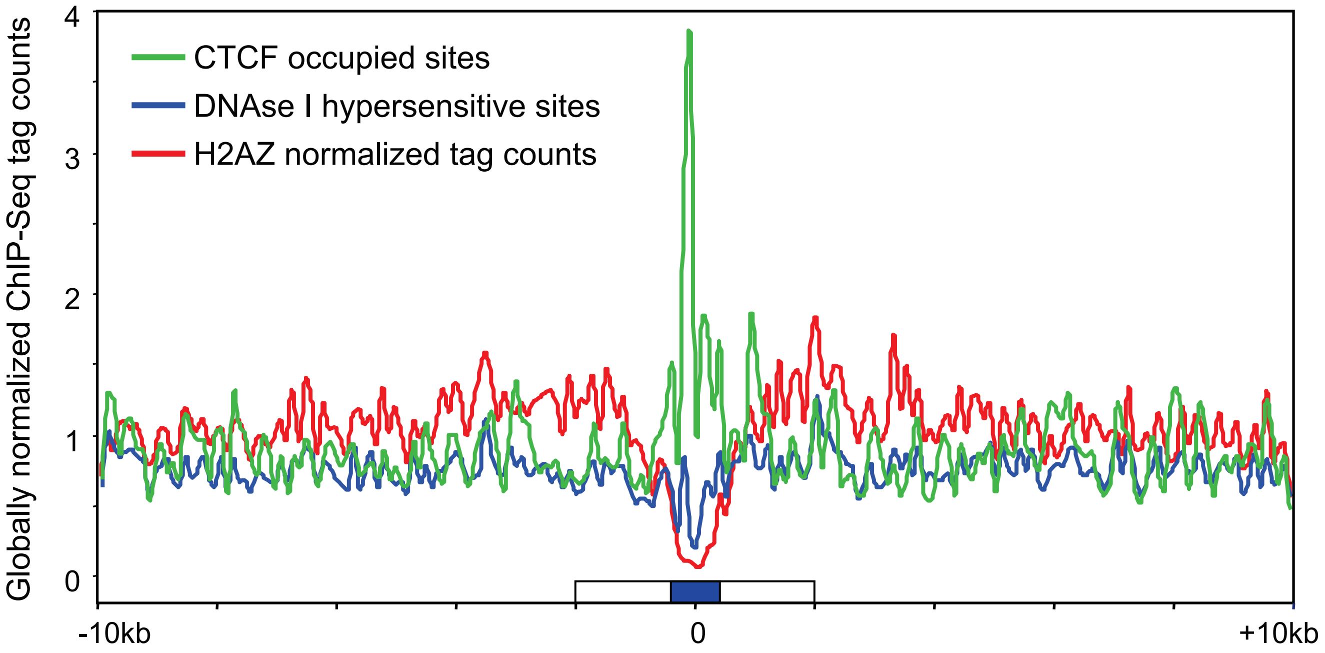

Figure S6. 1683 predicted human MAR genomic locations were aligned using the central positions of their AT rich cores. ChiP-Seq profiles were calculated over the MAR collection for association with the CTCF transcription factor, for DNAse hypersensitive sites and for the H2AZ histone variant. Tag counts were normalized globally and they are expressed as a fold change over the non-precipitated input DNA profile.
