# Supplementary material for: Molecular Characterization of a Human Matrix Attachment Region Epigenetic Regulator
Source: PLoS One. 2013 Nov 14;8(11):e79262. doi: 10.1371/journal.pone.0079262 (PMC3828356; doi:10.1371/journal.pone.0079262)
Supplement: Table S1 — Primer sets used to amplify portions of MAR 1–68 and MAR X-29. (PDF) [file pone.0079262.s007.pdf]

Table S1 Primer sets used to amplify portions of MAR 1-68 and MAR X-29.

**MAR 1-68**	**Forward primer (5' to 3')**	**Reverse primer (5' to 3')**
MAR 1-1652	GGATCC**CGGGGATCCTCTAGA**	TAGGAAGATCTC**CAATTGTCTCACTTTGTGGA**
1-910	GGATCC**CGGGGATCCTCTAGA**	AAGGAAGATCT**TGAAGAGCAGCATTATGAATAG**
864-1652	TACGCGGATCC**GAGAGATCATTAATTATAAT**	TAGGAAGATCT**CCAATTGTCTCACTTTGTGGA**
2444-3628 (end)	CGCGGATCC**ACTAGTAGAGTCTCGCTCTGT**	TAGGAAGATCT**CTAGATTATACCAACC**
2444-3020	CGCGGATCC**ACTAGTAGAGTCTCGCTCTGT**	TTGGAAGATCTT**CTTAACCTTTCCTAACCAC**
3000-end	CAACGCGGATCC**GTGGTTAGGAAAGGTTA**	TAGGAAGATCT**CTAGATTATACCAACC**
**MAR XS-29**	**Forward primer (5' to 3')**	**Reverse primer (5' to 3')**
1765-1996	**CTCCAGCCTAGGCAACAGAGT**GATATCATG	CGGGATAT**CACAGGGAAGACTATTCATACC**

BamHI and BglII sites on the forward and reverse primers for the MAR 1-68 PCR primers, or the restriction sites for EcoRV on both forward and reverse MAR XS-29 specific primers, are indicated by underlined text, while the bold text represents amplified MAR sequences.
