# Supplementary material for: Molecular Characterization of a Human Matrix Attachment Region Epigenetic Regulator
Source: PLoS One. 2013 Nov 14;8(11):e79262. doi: 10.1371/journal.pone.0079262 (PMC3828356; doi:10.1371/journal.pone.0079262)
Supplement: Table S2 — Primers sets used to amplify control cDNA sequences. (PDF) [file pone.0079262.s008.pdf]

Table S2 Primers sets used to amplify control cDNA sequences

**Luciferase**	**Forward primer (5' to 3')**	**Reverse primer (5' to 3')**
Luc 1-1652	CCCTCCCGGGCATGGAAGACGCCAAAAACAT	AGGGCCCGGGTACACGGCGATCTTTCCGCCCT
Luc 1-1884	CCCTCCCGGGCATGGAAGACGCCAAAAACAT	AGGGCCCGGGGGACCCCTCACACACAGTTCGCC
Luc 1-910	CCCTCCCGGGCATGGAAGACGCCAAAAACAT	AGGGCCCGGGATTTGTCAATCAGAGTGCTTTTGG
Luc 1-628	CCCTCCCGGGCATGGAAGACGCCAAAAACAT	AGGGCCCGGGGAGCGACACCTTTAGGCAGACCAGT
Luc 1-563	CCCTCCCGGGCATGGAAGACGCCAAAAACAT	AGGGCCCGGGCAATTGTCTTGTCCCTATCGAAGG
Luc 1-240	CCCTCCCGGGCATGGAAGACGCCAAAAACAT	AGGGCCCGGGAACGGACATTTCGAAGTACTCAGCG
**Utrophin**	**Forward primer (5' to 3')**	**Reverse primer (5' to 3')**
Utro 1-2000	CGGGGTACCCCAAGTATGGGGACCTTGAAGC	CGGAATTCCCAAACTTTTTCTTGGCCTCCACG

DNA fragments of 240-2000 bps in length, as indicated, served as negative controls to replace MAR portions of different lengths. Full-length MARs (1-68 and XS-29) or derivatives were replaced by sequences from the Utrophin (2kb) and Luciferase (1.6kb) cDNA. Underlined sequences are bases forming the restriction sites for SmaI on luciferase specific primers, KpnI and EcoRI on Utrophin specific primers
