# Supplementary material for: Molecular Characterization of a Human Matrix Attachment Region Epigenetic Regulator
Source: PLoS One. 2013 Nov 14;8(11):e79262. doi: 10.1371/journal.pone.0079262 (PMC3828356; doi:10.1371/journal.pone.0079262)
Supplement: Table S3 — Oligonucleotides containing transcription factor binding motifs. (PDF) [file pone.0079262.s009.pdf]

Table S3 Oligonucleotides containing transcription factor binding motifs

Putative TFs	Forward oligo (5' to 3')	Reverse oligo (5' to 3')
CEBP	GATCCAGTACT**GTTTGGGAAATT**CCATGGA	GATCTCCATGGAA**TTTCCCAAACA**GTACTG
Fast1	GATCCAGTACT**CATGTTCATTTT**CTCTAGA	GATCTCTAGAG**AAAATGAACATG**AGTACTGC
Hox	GATCCAGTACT**AATAATAAAATAC**CCGGGA	GATCTCCCGGG**TATTTTATTATT**AGTACTG
Gsh	GATCCAGTACT**CCCCTAATTCA**GACATGCA	GATCTGCATGTC**TGAATTAGGGG**AGTACTG
NMP4	GATCCAGTACT**GGGAAAAAAAT**CGTCGACA	GATCTGTCGACG**ATTTTTTTCCC**AGTACTG
SATB1	GATCCAGTACT**TTATTATAATA**TGTTAACA	GATCTGTTAACA**TATTATAATAA**AGTACTG
	BamH1	BglII

Binding motifs for CEBP, Fast1, Hox, Gsh, NMP4, and SATB1, as found in MAR 1-68 and predicted by the MatInspector software, are indicated in bold letters. Underlined sequences on the forward and reverse primers are bases forming the restriction sites for BamHI and BglII, respectively.
