# Supplementary material for: Molecular Characterization of a Human Matrix Attachment Region Epigenetic Regulator
Source: PLoS One. 2013 Nov 14;8(11):e79262. doi: 10.1371/journal.pone.0079262 (PMC3828356; doi:10.1371/journal.pone.0079262)
Supplement: Table S4 — Quantitative PCR primer sets for GFP, GAPDH and eEFIA. (PDF) [file pone.0079262.s010.pdf]

Table S4 Quantitative PCR primer sets for GFP, GAPDH and eEFIA

**Gene**	**Forward oligo (5' to 3')**	**Reverse oligo (5' to 3')**
GFP	AGCAAAGACCCCAACGAGAA	GGCGGCGGTCACGAA
GAPDH	CGACCCCTTCATTGACCTC	CTCCACGACATACTCAGCACC
eEFIA	TCCACTTGGTCGCTTTGCT	CTT CTT GTC CAC AGC TTT GAT GA

The same pair of GFP specific quantitative PCR primers was used to quantify GFP transgene copy number and mRNA levels. Primers to two housekeeping genes, GAPDH and eEFIA were used as internal controls for normalization to quantify GFP and mRNA copy numbers, respectively.
